# Using Artificial Intelligence to Enhance Myelodysplastic Syndrome Diagnosis, Prognosis, and Treatment

**DOI:** 10.3390/biomedicines13040835

**Published:** 2025-03-31

**Authors:** Fabio Stagno, Giuseppe Mirabile, Patricia Rizzotti, Adele Bottaro, Antonio Pagana, Sebastiano Gangemi, Alessandro Allegra

**Affiliations:** 1 Division of Hematology, AOU Policlinico “G. Martino”, Department of Human Pathology in Adulthood and Childhood “Gaetano Barresi”, University of Messina, Via Consolare Valeria 1, 98125 Messina, Italy; giuseppe.mirabile@polime.it (G.M.); patricia.rizzotti@polime.it (P.R.); adelebottarp15@gmail.com (A.B.); antoniopagana94@gmail.com (A.P.); aallegra@unime.it (A.A.); 2 Allergy and Clinical Immunology Unit, Department of Clinical and Experimental Medicine, University of Messina, Via Consolare Valeria, 98125 Messina, Italy; gangemis@unime.it

**Keywords:** artificial intelligence, machine learning, myelodysplastic syndromes, MDS, diagnosis, flow cytometry, prognostic scoring system, minimal residual disease, blood biomarkers

## Abstract

Myelodysplastic syndromes represent a group of hematological neoplastic diseases caused by defective stem cells causing cytopenia and abnormal hematopoiesis. More than 30% of myelodysplastic syndrome cases develop into acute myeloid leukemia. An analysis of bone marrow samples, peripheral blood smears, multiparametric flow cytometry data, and clinical patient information is part of the current, time-consuming, and labor-intensive work up for myelodysplastic syndromes. Nowadays, clinical biomedical research has been transformed by the advent of artificial intelligence, specifically machine learning. Artificial intelligence (AI) can improve risk assessment and diagnosis, as well as boost the precision of clinical outcome prediction and illness classification. Algorithms based on artificial intelligence may be potentially helpful in discovering new needs for myelodysplastic syndrome-affected patients, choosing treatment and assessing minimal residual disease. In this review, we seek to identify the primary mechanisms and uses of artificial intelligence in myelodysplastic syndrome, pointing out its advantages and disadvantages while discussing the possible benefits of using AI pipelines in a therapeutic setting.

## 1. Introduction

### General Information on Myelodysplastic Syndromes

The growth of a clonal hematopoietic stem/progenitor cell through the acquisition of recurrent genetic alterations represents the cause of myelodysplastic neoplasms or myelodysplastic syndromes (MDSs). These conditions are characterized by variable degrees of hematopoietic cell morphologic dysplasia, immunophenotypic abnormalities both of blasts and maturing myeloid elements, and ineffective hematopoiesis that leads to peripheral blood (PB) cytopenias. More than 30% of MDS cases eventually advance to acute myeloid leukemia (AML) [1]. An accurate diagnosis and subclassification of MDS needs the integration of clinical, morphologic, and genetic data. Hence, these findings influence risk assessment and treatment planning [2]. Cellular dysplasia, blast count, cellularity, monocytes, ring-sideroblasts, and a few other characteristics have all been evaluated in stained bone marrow (BM) samples by light microscopy for decades to improve the diagnosis of MDS. The World Health Organization’s (WHO) categorization system, which incorporates morphology, blood count, and cytogenetics, has divided MDS into subgroups [3]. Even a highly skilled hematopathologist may find it challenging to establish an accurate diagnosis in some situations due to the quantity and complexity of features used for diagnosis as well as the subjectivity of evaluation in bone marrow assessments. Recently, MDS cells have been further characterized because of genetic studies. Several genes including TET2, ASXL1, SF3B1, SRSF2, DNMT3A, and RUNX1 were found to be altered [4]. The association between morphology and gene mutations is still poorly understood, with a few notable exceptions, such as the existence of ring-sideroblasts in SF3B1-mutated MDS or the megakaryocyte dysmorphology in 5q-deleted MDS. Moreover, it is challenging to forecast the course and severity of the disease because comparable patient cohorts might show a different range of clinical outcomes from benign with just sporadic blood cell transfusion requirements to a rapid development into AML [5,6,7]. All the aforementioned factors represent the challenge to achieve quick and accurate MDS diagnosis and prognosis assessment that can drive the best therapeutic action.

## 2. Methods

### 2.1. Use of Artificial Intelligence in Hematology

The technology that allows computers and other devices to mimic human learning, comprehension, problem-solving, decision-making, creativity, and autonomy is known as artificial intelligence (AI). The use of sophisticated AI models and algorithms in clinical laboratories is a new and unavoidable stage in the development of laboratory medicine, since a significant amount of laboratory data can be used to generate diagnostic and prognostic panels unique to diseases. In the fields of hematology, cytology, and histopathology, machine learning (ML) has made it feasible to analyze large amounts of unstructured digital images and organized numerical data [8,9,10]. The intricacy of myeloid neoplasm classification has made it difficult to integrate genomic data with conventional morphological criteria. Furthermore, gene expression and methylation profiles can also reveal details about the pathophysiology of the disease, which complicates matters. It may be possible to enhance existing categorization systems and successfully combine various diagnostic modalities with new machine learning technology. Soon, the use of machine learning and sophisticated statistical techniques on sizable patient populations might improve classifications by developing objective and reliable illness categories that were previously unknown [11]. Supervised and unsupervised learning are the two primary forms of ML that have been investigated in hematology contexts. As for the distinction between supervised and unsupervised learning models, supervised learning involves training the model on a labeled dataset, which means that every training example has an output label. The objective is to teach the model a mapping from inputs to outputs, since it can forecast the output given fresh unobserved data. Neural networks, logistic regression, linear regression, and support vector machines are examples of common algorithms. This approach is utilized for tasks such as regression and classification. In contrast, unsupervised learning uses an unlabeled dataset—that is, data without any predetermined labels or categories—to train the model. Finding hidden patterns or intrinsic structures in the data is the aim, and popular techniques for this purpose include dimensionality reduction (e.g., PCA, t-SNE) and clustering (e.g., K-means, hierarchical clustering). Data visualization and anomaly detection are some of the possible means to employ this approach [12].

Reinforcement learning (RL) is a third type of machine learning that has been introduced lately by developing a learning model that includes three basic steps, namely state, action, and reward. The state represents a certain condition, the action is the response to the condition, and the reward is feedback [13]. Supervised learning creates an algorithm that links variables and pre-established labels. The performance of the algorithm is then evaluated by forecasting results in separate validation datasets. Conversely, unsupervised learning finds relationships and patterns between variables that are unlabeled (without a clear goal). Clusters created by the produced algorithms are then frequently assessed for validity in supervised learning [14]. The rate of correct predictions overall, recall (or sensitivity), specificity, the accuracy of the model in positive cases, precision (or positive predictive value), the rate of actual correct predictions in positive cases and negative predicting value, and the capacity to accurately identify negative cases are some of the metrics used to express the adequacy of machine learning models as the sufficient criteria needed to perform tests [15].

### 2.2. Use of Artificial Intelligence in Myelodysplastic Syndromes

**Figure 1:**
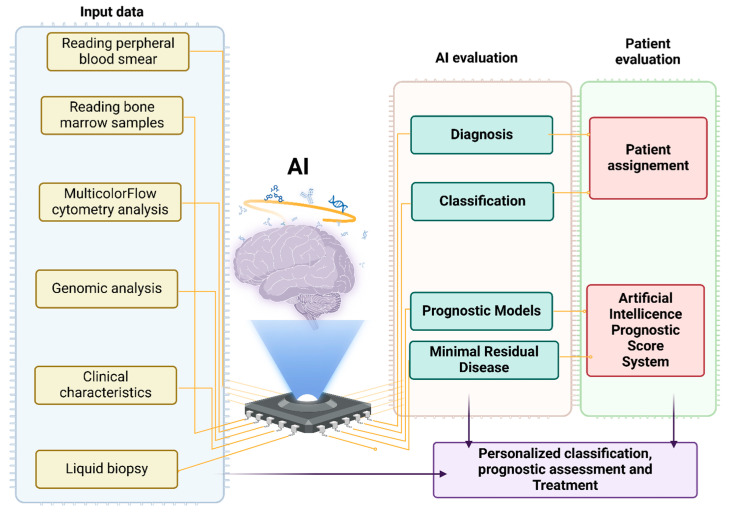
Management of patients with MDS using artificial intelligence.

Diagnosing instances with modest cytopenias and subtle dysplastic alterations can be challenging even among skilled hematopathologists, and subjectivity and inter-scorer variability may exist [16]. In many cases, qualifying dysplasia cannot be identified clearly, and indefinite conclusions may be drawn despite persistent cytopenias. In this case, patients are categorized into two streams, with idiopathic cytopenia of undetermined significance (ICUS) and clonal cytopenia of undetermined significance (CCUS), when clonality is evident [17,18]. The viability of ML applications in myeloid neoplasia (MN), particularly in MDS, has been demonstrated in recent years by an increasing amount of data. Many studies concentrated on supervised learning applications to identify biomarkers that might predict outcomes and diagnostic indicators aiding in clinical decision-making [19] (Figure 1). AI was used in studies to diagnose MDS utilizing data from multicolor flow cytometry (MFC), bone marrow cells (BMS), and peripheral blood cells (PBS).

## 3. Results

### 3.1. Use of Artificial Intelligence in Reading Peripheral Blood Smears

Light microscopy interpretation of peripheral blood smears is laborious, time-consuming, and prone to diagnostic errors due to substantial inter-observer variability [20]. In the last ten years, numerous attempts have been made to automatically identify the types of cells present in blood smears. At first, researchers created algorithms to identify nuclear segmentation, leukocytes, or red blood cells. After it was shown that AI and its application to the morphological classification of blood cells might reduce the interobserver variability and subjective human error, they began to address the detection of abnormal leukocytes, including different types of leukemic cells [21,22,23,24,25,26,27,28,29,30,31,32,33]. However, several technological issues must be solved before AI use. Large annotated datasets are really used to teach modern AI systems. Digital slide scanners are employed for image acquisition, followed by slide and image de-identification, quality and resolution evaluation, digital image storage, and annotation and validation of the cells of interest [34]. To objectively determine a set of categorization parameters to make final decisions, several ML techniques employ feature extraction algorithms. They consist of the cytoplasm and cell nuclei’s texture, color, and shape characteristics. Since convolutional neural networks (CNNs) have proven particularly well suited to image analysis, they are typically used to create successful deep learning models for medical images [35]. Currently, the Food and Drug Administration approved four digital morphology commercial analyzers that use AI-based software as a medical device (SaMD) as Class II medical devices [36,37,38,39,40,41]. Commercial AI analyzers can accurately identify objects and classify common WBC, red blood cells, and platelets. However, they have major gaps in less frequent cell kinds, which leads to the incorrect categorization of aberrant cell types. Additionally, the sensitivity of current commercial AI morphology analyzers is inadequate for more complicated diagnosis such as lymphoproliferative disorders, myeloproliferative neoplasms, and myelodysplastic syndromes. Lastly, data from research using AI models trained on private datasets are not shared, and this prevents reliability verification and a broader adoption of such models, even if peripheral blood film cellular morphology does not differ by age, gender, or ethnicity [36,37,38,39,40,41]. Nonetheless, significant advancements have been made in the development of AI algorithms together with categorization systems based on whole-slide imaging. A whole blood smear can be analyzed quickly thanks to this capacity for large-scale computation and analysis. The detection of MDS was investigated. On 8940 single-cell validation images, Kimura et al. classified blood cells into 17 classes with an accuracy of 92.05% [42]. Segmented neutrophils, band neutrophils, metamyelocytes, myelocytes, promyelocytes, blasts, eosinophils, basophils, lymphocytes, variant lymphocytes, platelet aggregations, big platelets, megakaryocytes, smudge cells, and artifacts were among the classes. This included 3261 peripheral blood smears with 703,970 single-cell pictures, including 1165 from patients with hematological diseases, making it the largest private dataset. Of these, 695,030 photos were utilized to train the CNN-powered deep learning system. This approach achieved a sensitivity of 88.4% and a specificity of 92.7% when used to categorize 20 distinct types of blood cell abnormalities. In this way, an automated diagnosis of MDS or aplastic anemia (AA) was carried out. A decision-making algorithm (XGBoost) was fed with 114 image pattern parameters from smears of MDS and AA patients, and it automatically analyzed the type and extent of normal and dysmorphic images with 96.2% sensitivity and 100% specificity [42]. However, the DL tended to misclassify big platelets as thrombocyte aggregations, band neutrophils as metamyelocytes, promyelocytes as myelocytes, segmented neutrophils as band neutrophils, and lymphocytes as variant lymphocytes. Thus, this system remains to be adjunctive in its nature.

### 3.2. Use of Artificial Intelligence in Reading Bone Marrow Samples

Naturally, BM smears are thought to be necessary for the diagnosis of MDS. They offer a thorough understanding of the morphology, cytogenetics, and composition of cells. On BM smears, dysplasia and blasts less than 20% are characteristic of MDS, and only when at least one lineage shows 10% dysplasia, MDS with dysplasia can be diagnosed [43]. Due to its complexity, BM has been the subject of few investigations [44,45,46]. Compared to peripheral blood, a microscopic field includes more information because BM specimens contain a wide variety of progenitor cell types with different stages of maturation. Furthermore, although peripheral blood examination mostly concentrates on cell counting, BM smear examination necessitates morphological evaluation in clinical settings. These obstacles delayed the use of ML technology in the diagnosis of BM diseases and hindered the development of an automated analysis of BM smears. A CNN-derived machine learning model that automatically identifies dysplasia from pictures of BM aspirates was reported by Lee and colleagues [47]. For the program’s examples, they used pictures of widely dispersed regions with nucleated cells. In addition, they identified the borders of 946 cells and divided 8065 cells into eight categories, namely blasts, dysplastic megakaryocytes, dysplastic granulocytes, dysplastic erythrocytes, normal granulocytes, and normal megakaryocytes. The model was trained to recognize and categorize various cells. Ten percent of the cell pictures were used for testing, ten percent for validation, and eighty percent for training. With an area under the curve (AUC) ranging from 0.945 to 0.996, the model demonstrated an exceptional AUC for the identification of dysplasia in every cell type [47]. However, it is crucial to underline that Lee and colleagues’ model was unable to measure the proportion of dysplasia despite its remarkable capacity to detect the presence of dysplastic cells in three distinct lineages. Additionally, Lee’s model has not been evaluated externally. An AI system that evaluates the presence of dysplastic cells on BM smears and the prediction for dysplasia-decreased granules (DGs) was used by the authors of a separate study [48]. Using an initially created cell detector, they clipped each cell in field photos taken from BM smears from patients with MDS or non-MDS conditions. Each cell was labeled by two morphologists. A four-point rating system was used to assess the degree of dysplasia. The classifier was subsequently built by the authors using the dataset of labeled photos. A deep neural network pre-trained using real-world images served as the foundation for both the detector and the classifier. The morphologists identified 134 DGs from 1797 acquired and labeled pictures. Then, they conducted a five-fold cross-validation to assess the classifier’s performance. The sensitivity, specificity, and accuracy were 85.2%, 98.9%, and 98.2%, respectively, but DG1 was not included in the procedure. To analyze BM smears for quicker diagnosis and disease monitoring, additional research was conducted to create a deep learning model (BMSNet). In a recent study, 122 BM smears were imaged and split into three groups, namely competition, validation, and development [49]. There were 17,319 annotated cells from 291 high-resolution images in the development cohort. Each patient in the competition cohort and validation group had a total of 20 pictures. The eight annotation categories used in this investigation were erythroids, blasts, myeloids, lymphoids, plasma cells, monocytes, megakaryocytes, and unidentified cells. A human–machine competition was held with six visiting staff members, and the FCM’s results were considered the ground truth. Six-fold cross-validation indicated that the average bounding box prediction precision in the development cohort, excluding categorization, was 67.4%. In many categories, BMSNet’s recall and precision were comparable to those of the hematologists. The AUC of BMSNet was lower than that of the pathologists but greater than that of the hematologists in identifying over 5% of blasts in the validation cohort. The hematologists’ and pathologists’ AUCs were comparable and greater than BMSNet’s in identifying over 20% of blasts. A subsequent investigation revealed that the MDS cases were responsible for the performance discrepancy [49]. In conclusion, this deep learning model can help hematologists to analyze BM smears. However, skilled hematologists are still needed for a thorough morphological interpretation. In fact, this study has limitations that must be noted. The analyzed images were first taken by skilled experts who had to modify the brightness and focus length. To minimize the operator effect, an automatic slide scanner was used. Second, the authors compared the system’s performance with that of just six visiting employees. Additional specialists should be compared to assess the system’s effectiveness.

Lastly, a study assessed control people, MDS/MPN patients, and MDS patients [49]. Five hundred tiles were examined in total. Brück and colleagues used the uniform manifold approximation and projection (UMAP) technique, which is most frequently used for analyzing single-cell transcriptomic data to map each tile onto a two-dimensional space after extracting features using the popular VGG16 and Xception CNN. This unsupervised analysis was carried out. Tiles with comparable picture feature profiles are situated adjacent to one another in this representation’s two-dimensional space. A visual inspection of the tiles within each of the five clusters revealed information about their contents. High concentrations of lipid droplets, red blood cells, stroma, hypocellular tiles, and hypercellular tiles were seen in these clusters. After averaging feature profiles for tiles in the same sample, Brück and colleagues used UMAP to reproject the samples in two dimensions and then unsupervised clustering. Once more, five clusters appeared. In addition to being homogeneous, healthy participants were also very different from the four MDS clusters, forming a unique cluster. Specific MDS WHO subtypes were enriched in each of the four clusters. The WHO subtypes and MDS sample categories, however, only partially overlapped. This is not surprising, considering that the WHO subtypes are determined by cytogenetics, blast percentage, blood cell counts, and bone marrow cytomorphology. Brück and colleagues proceeded to use supervised analysis to train statistical models based on elastic net-regularized regression to predict various tumor and patient characteristics based on image information. Some traits, including chromosome 7 monosomy, 7q deletion, and mutations in TET2, ASXL1, and STAG2, proved to be very predictive based on morphologic aspects. This indicates that there are more connections between morphologic characteristics and mutations in MDS than previously believed. By using only features taken from stained slides, Brück and colleagues were also able to predict the risk stratifying IPSS-R score (Revised International Prognostic Scoring System), overall survival, and progression to acute myeloid leukemia (AML) with good accuracy. Of interest, the model that best predicted progression to AML incorporated traditional IPSS-R scores with histopathological markers. With an accuracy of 0.81, the model was able to distinguish between patients with MDS and those with MDS/MPN. Predicted MDS samples were hypoplastic with more stromal involvement, as expected, according to analysis of direct cell and picture segmentation and classification [50].

### 3.3. Use of Artificial Intelligence in Flow Cytometry Analysis of MDS Samples

Flow cytometry (FC) can supplement the primary morphological evaluation, even though a cytological assessment of BM smears remains the first step in the search for potential hemopoietic dysplasia. This is particularly true in borderline cases where immunophenotypic aberrations can either confirm or rule out an MDS diagnosis. Other authors have confirmed the ELN iMDS flow proposal that the four-parameter Ogata score [51] may be utilized for an initial evaluation of multicolor FC (MFC) dysplasia [52]. This score is determined by the following factors: low granulocyte scatter (granulocyte/lymphocyte SSC ratio ≤ 6), aberrant CD45 expression on CD34+ blasts, the fraction of B-cell precursors inside CD34+ cells (<5%), and the frequency of myeloid CD34+ myeloid progenitors (MPs) (>2%). Though feature-based analysis is the gold standard for analyzing imaging flow cytometry (IFC) data, the rigidity of picture segmentation can make it very difficult, time-consuming, and ineffective to generate acceptable and effective analysis strategies. In terms of improving diagnosis, AI-based flow cytometry analysis demonstrated encouraging outcomes. Several studies investigated dyserythropoiesis using image analysis [53] and employed multi-parameter FC or real-time deformability cytometry inputs to distinguish between normal and MDS bone marrow [38,53,54,55]. To this address, IFC with the ImageStream ^®^X MKII (ISX) can combine the high-resolution imaging capabilities of microscopy with the statistical robustness and high-throughput data collecting capacity of traditional MFC in a single system. Twelve images—two brightfield (BF) and ten fluorescents—can be taken simultaneously from each individual cell that moves through the system thanks to the ISX. In addition to using feature-based algorithms and artificial intelligence to quantify morphometric changes that can be standardized and automated, the acquired image data from thousands of cells can be analyzed using conventional MFC gating strategies to identify phenotypical markers [56].

Trying to improve MDS diagnosis and classification, Clichet and colleagues proposed a novel method that combines AI with multiparametric FC [57]. An elastic net approach was used in their machine learning model and applied to a group of patients who were suspected of having MDS. The Boruta algorithm was employed for feature selection, and the study looked only at flow cytometry parameters. The most significant indicators for MDS diagnosis were determined to be the ratio of granulocyte/lymphocyte SSC peak channels, total hematogone ratio, percentage of CD34+ B-cell progenitors among all CD34+ cells, and the percentage of CD34+ myeloid progenitors. With outstanding specificity, the AI-assisted MDS prediction score exhibited superior sensitivity to the Ogata score. Its excellent performance was further confirmed by an external validation cohort of patients. Notably, both high-risk and low-risk MDS may be accurately diagnosed using this model, and it indicated a linear transition between these stages by showing a progressive evolution of the prediction score from clonal hematopoiesis of indeterminate potential (CHIP) to high-risk MDS [57]. A computational approach for FC diagnosis in suspected MDS was also showed by Duetz et al. [58]. Patients in the study cohort included age-matched controls with non-neoplastic cytopenia and patients with MDS. A standardized panel of six tubes was used to capture FC data, and quality control and outlier exclusion were part of the preprocessing step. A random forest ML classifier and the flow-self organizing maps (FlowSOM) technique for cell population detection were included into the diagnostic workflow. Expert-analyzed FC scores, including the Ogata score and the integrated flow cytometry score (iFS), were compared to the procedure. With processing times lowered to less than two minutes per patient, the computational processes scored better than the scores in terms of accuracy, objectivity, and time investment. A single-tube computational method was also created and in the external validation cohort, it demonstrated even greater sensitivity and specificity. Furthermore, the computational workflow demonstrated that the diagnosis of MDS was significantly influenced by specific cellular characteristics, especially those of erythroid and myeloid progenitors [58].

In another investigation [59], FlowSOM was able to detect six subpopulations of erythropoietic precursors (EPs). MFC data from list-mode files of individuals with MDS and non-clonal anemia were examined to determine how this program would aid in the characterization of erythropoiesis alterations. Eighteen more EP categories that were absent from the combined normal BM samples were found using unsupervised FlowSOM analysis. Many of them featured minor, unanticipated, and unreported changes inside scatter properties and the expression of the CD36 and/or CD71 antigens. In samples from patients with MDS, three patterns were found as follows: i) EPs with abnormal proliferating precursors and decreased proliferation, ii) EPs with a normal proliferating fraction and maturation defects in late precursors, and iii) EPs with a reduced erythropoietic fraction but normal patterns indicating that erythropoiesis was less affected. Furthermore, an examination of successive samples from a patient with MDS undergoing treatment revealed that aberrant subsets decreased during azacytidine treatment and that allogeneic hematopoietic stem cell transplantation brought the samples close to normal [59]. Subtle changes in erythropoiesis that are not visible by cytology or FCM supervised analysis are revealed by an unsupervised clustering analysis of MFC data.

Other research tried to examine some aspects of MDS patients and utilize them to reach a particular diagnosis. The use of AI software to detect binucleated erythroblasts (BNEs) and distinguish them from doublet events and other non-BNE images was investigated [60]. They demonstrated similarity between the AI-derived BNE frequencies and those previously obtained using feature-based machine learning (FBML). Crucially, each individual cell’s picture from patients and controls was physically inspected to visually identify and confirm the authenticity of the BNEs.

Given the limited resources in our healthcare system and the need to apply less labor-intensive approaches, it is feasible that an AI-driven strategy will both improve and speed up work procedures in clinical laboratories. Future research might incorporate the patient’s clinical history with important biochemical test parameters helping the interpretation of blood smears. To further remark on the type of anemia, creatinine and liver function tests, iron studies, vitamin B12, and folate levels can be combined (Table 1).

### 3.4. Use of AI in the Differential Diagnosis of MDS with Aplastic Anemia and Acute Myeloid Leukemia

Differentiating MDS from leukemia and aplastic anemia (AA) is another issue related to diagnosis, since AA and MDS, especially hypocellular MDS, might share comparable clinical and hematological characteristics. Hematologic analysis, bone marrow biopsy, cytogenetics, and FC are examples of modern diagnostic techniques. Both diseases have ineffective hematopoiesis, which is nonspecific. Cytogenetic abnormalities, once believed to be reliable, are no longer specific to MDS. Despite FC’s increasing popularity, diagnosing MDS is challenging due to its single marker usage and limitations in recognizing erythroid cancers. In addition to their similarities, the low specificity of many indications makes it challenging to differentiate MDS from AA. A deep learning approach for the automatic diagnosis of MDS and the differentiation between AA and AML based on BM smears was reported by Wang et al. [61]. Data from the American Society of Hematology (ASH) Image Bank was used to train the CNN-developed model, while data from clinical findings was used for external validation. Training and testing datasets were created by randomly dividing the ASH data in a 7:3 ratio. For every model, three distinct epochs (30, 50, and 200) were employed. This established how many times the learning model was shown the training data. The patient’s MDS status (two classifications) and whether they had AA, MDS, or AML (three categories) were the model’s two output layers. An outcome weight of 1:9 and an epoch of 200 produced the optimum model training effect. The model demonstrated strong performance metrics in separating MDS from non-MDS and in differentiating between MDS, AA, and AML upon external validation. Overall, using bone marrow smear images, the image net pretrained model offered clinicians a quick and precise way to distinguish between AA, MDS, and AML.

## 4. AI and Prognosis Evaluation in MDS

Elshoeibi et al. recently conducted a thorough evaluation examining the potential use of AI in MDS patients. The aim of this work was to investigate the possible uses of machine learning algorithms in the diagnosis of MDS and the development of differential diagnoses with other hematologic disorders. In addition, we also looked into the potential prognostic application of AI in MDS patients and its use to address other unmet needs of MDS patients [62].

Because the clinical course and outcome of MDS patients can vary, risk-adapted classification models are essential for determining the best treatment. The most widely used method for estimating survival and evaluating disease-related risk is the International Prognostic Scoring System, Revised Version (IPSS-R), which is based on hematological and cytogenetic characteristics. However, predictive data at the individual patient level are not provided by the IPSS-R [63,64]. Several molecular models have been proposed to represent different biological MDS subgroups and improve treatment approaches [65,66,67]. To categorize patients with MDS into six risk groups, the International Working Group for Prognosis in MDS introduced the Molecular IPPS (IPSS-M) model, which uses clinical characteristics, cytogenetic abnormalities, and molecular information for 31 genes [68]. Compared to earlier models, including the original IPSS, IPSS-R, and the World Health Organization’s (WHO) classification-based Prognostic Scoring System (WPSS), the IPSS-M has demonstrated superior prognostic power, allowing for an improved prediction of leukemia transformation and overall survival (OS) [69,70,71]. Additionally, information about the likelihood of response to treatments, including hematopoietic stem cell transplantation (HSCT) and hypomethylating drugs (HMAs), was better provided by the IPSS-M [72,73]. Based on traditional clinical criteria, the Artificial Intelligence Prognostic Scoring System for MDS (AIPSS-MDS) performed better than the IPSS-R. A study compared the AIPSS-MDS models with earlier score systems. This retrospective analysis recruited molecular and clinical data from patients with MDS and patients with MDS/myeloproliferative neoplasms. The IPSS-M prognostic discrimination was superior to the IPSS-R model and was comparable to the AIPSS-MDS model. According to a prognostic power evaluation, 12.6% of patients were upstaged and 5% were downstaged following re-stratification from IPSS-R to IPSS-M, considering simplified low- and high-risk groupings for therapeutic care. On the other hand, no patients were downstaged and 51% of the low-risk cohort were reclassified as high-risk by the AIPSS-MDS. Compared to the IPSS-R, EuroMDS, and MLL models, the IPSS-M and AIPSS-MDS models offer more precise survival predictions [74]. Therefore, the AIPSS-MDS model might be a good choice for determining the risks for every patient with MDS, particularly at facilities with limited resources where genetic testing is not a common clinical procedure. MOSAIC, another innovative AI-based system for multimodal analysis, classification, and individualized prognostic assessment in rare malignancies, was used in a different study [75]. The clinical and genetic features of 4427 MDS patients were evaluated and split into training and validation groups and were integrated and imputed using deep learning techniques. In contrast to the traditional hierarchical Dirichlet process (HDP), clustering was carried out by combining the Uniform Manifold Approximation and Projection for Dimension Reduction 1 Hierarchical Density-Based Spatial Clustering of Applications with Noise (UMAP 1 HDBSCAN) techniques. For survival prediction, linear and AI-based nonlinear methods were contrasted. By incorporating the clinical models into distributed infrastructure, explainable AI (Shapley Additive Explanations method [SHAP]) and federated learning were utilized to enhance the clinical models’ performance and interpretation. With a better average silhouette coefficient in relation to HDP and a greater balanced accuracy in cluster classification by random forest, UMAP 1 HDBSCAN clustering produced a more detailed patient stratification. The reference prognostic tool for MDS and traditional statistical methods are outperformed by AI methods for survival prediction. As a result, MOSAIC offers a reliable and understandable paradigm for maximizing rare cancer classification and prognostic evaluation [75]. Regardless of clinical–morphological characteristics, unsupervised ML played a crucial role in identifying functional objective molecular clusters in MDS. In fact, 14 different molecular clusters were identified using ML models when applied to a dataset of 3588 patients with MDS and subsequent AML. Different genetic characteristics, such as karyotype and the frequency of molecular mutations and their combination, were used to describe each cluster [76]. After adapting to the new IPSS-M, the ML model was also able to handle clinical implications in terms of overall survival and response to treatment. The architecture of AML and MDS was resolved using multimodal characteristics, which revealed convergent molecular and functional pathways for potential future therapeutic uses [76]. AI models may also be helpful in forecasting how therapies will work. The current standard of therapy for patients with intermediate-to higher-risk MDS is to use the hypomethylating drugs (HMA) decitabine and azacitidine. HMA prevents AML transformation, prolongs survival, and improves cytopenia [77,78]. Addressing MDS risk classification, Nazha et al. developed a trained and unsupervised personalized prediction model based on genetic alterations, laboratory results, 2016 WHO subtypes, and demographics [79]. The concordance index, which is the same as the area under the receiver operating characteristic curve, is the most often used statistical indicator to assess survival results. When compared to the Revised International Prognostic Scoring System, the model’s C-index for overall survival prediction was higher at 0.74. By examining serial blood counts over the first ninety days of treatment, an ML model was also created to evaluate the response to HMA. After three months, the model was able to forecast the HMA response based on hemoglobin, platelets, RDW, and monocyte count. An independent cohort was used to validate this training cohort-based algorithm [80]. Using a real-world cohort of more than 1000 AML and MDS patients with more than 5000 MFC data records on bone marrow samples, another study used two AI approaches to create an MFC interpretation system for minimal residual disease (MRD) identification [81]. The algorithm’s good result prediction in the post-induction setting revealed its high clinical validity. The authors showed that the AI-developed methods could complete the classification task in about 7 s with an accuracy of almost 90% on MRD detection on AML and MDS. Furthermore, the outcomes predicted by the AI algorithms in the postinduction setting showed a high prognostic relevance. By examining early alterations in patients’ blood counts, study created a model to evaluate HMA responsiveness more quickly [80]. A model that evaluated patients’ response to therapy 90 days after initiation using serial blood counts was developed using data from three universities. A training cohort of 424 patients from two institutions was used to create the model and an independent cohort of 90 patients was used for validation. In the training/testing group and the validation group, the final model’s area under the receiver operating characteristic curve was 0.79 and 0.84, respectively. All the patients used for training and validation, however, originate from MDS referral centers rather than the community because of the data needs. The training data may have been biased toward MDS individuals with more aggressive illness, more comorbidities, or other unfavorable prognostic factors because of the removal of such patients. On the other hand, enrolling patients in clinical trials may result in the selection of more fit individuals. However, verifying the model in different practice situations will be a key future goal.

## 5. AI and Emotional Needs of MDS Patients

Determining the informational, functional, and emotional demands of patients with MDS and how these evolve throughout the course of the disease is a major issue in MDS management. When patients or caregivers are unable to obtain enough information or emotional support from doctors, they may resort to other resources, such as internet discussion boards. Social media posts were used in earlier studies to learn about the requirements and viewpoints of AML and MDS patients who were not eligible for intense chemotherapy. To find the overarching themes, fewer posts were examined by hand [82]. To determine the unmet needs of MDS patients and their caregivers, a study performed a social listening analysis of publicly accessible internet forums [83]. The authors categorized post categories using AI and natural language processing (NLP) into seven main reasons for online interaction, including clinical, emotional, treatments, transplant, education and logistics, physical, and diet and lifestyle. Results showed that patient needs varied based on the nation of residency and available treatments. Educational initiatives could provide Spanish patients for whom active treatments are still working a more comprehensive understanding of therapy and palliative care options throughout the end of life. A more emotional support throughout latter stages of treatment and treatment discontinuation might be beneficial for patients in the USA, UK, and Canada. Eligible patients may also benefit from targeted education regarding transplant adverse effects and predicted results (quality of life, efficacy, etc.). AI reduced the likelihood of bias and allowed for the organization and analysis of a larger dataset by organizing and analyzing the massive amount of data. The results of this analysis added to and elaborated on earlier findings of patient requirements from the analysis of internet forums by highlighting additional difficulties that patients and caregivers may encounter.

## 6. Conclusions

### 6.1. Future Perspectives

These models and innovative technology techniques are of crucial importance in clinical practice [84,85]. In this context, AI has enormous potential in the medical field [86]. By integrating AI, it may be possible to eliminate operator-dependent errors and simplify the intricate interpretation of molecular testing. Nonetheless, there are further uses for AI in MDS patients. To improve the effectiveness of therapeutic medications, recent research on clinical pharmacology has called for the development of innovative, promising, and eco-friendly instruments [87,88]. Many attempts have been made to create various strategies to lessen the use of potentially harmful or deleterious organic solvents to accomplish this goal. One of the most innovative technologies for creating affordable therapeutic medications is the effective synthesis of solid-dosage oral formulations utilizing environmentally friendly supercritical solvents. One important factor that needs to be assessed before process design is drug solubility. Decitabine is used to treat individuals with MDS and is a member of the antimetabolite class of chemotherapeutic drugs [89]. Due to the high expense of experimental research, the prediction of drug solubility using mathematical models and artificial intelligence has gained attention in recent years. The study’s goal was to create a variety of ML-based models to determine the anti-cancer medication decitabine’s ideal solubility and assess how temperature and pressure affected it. The authors employed three ensemble methods—random forest (RFR), extra tree (ETR), and gradient boosted regression trees (GBRTs)—to create models on a limited dataset. Optimal hyper-parameters were discovered after testing various combinations [90]. In conclusion, the AI models may be used as supplemental resources to help pathologists and hematologists to diagnose and treat MDS more quickly and affordably. These models have not been created and tested sufficiently to play the role of skilled pathologists and hematologists.

### 6.2. Challenges of Implementing AI Tools in Clinical Settings

In addition, there are some issues that must be solved. The proper therapeutic use of AI invariably mentions issues of data use and individual autonomy, which need to be properly handled.

The quality of data is one of the main technological constraints that machine learning algorithms must contend with in the medical field. Inaccurately labeled or missing data plague laboratory system information by limiting the maximum performance that any algorithm can attain. The cost of the computing infrastructure and the hiring of the staff with the necessary computational skills to create, implement, maintain, and upgrade machine learning algorithms and the software tools required to run them represent yet another technical and financial obstacle. This all affects issues with inadequate model standardization and discrepancies in findings from various labs and research facilities. Guidelines are currently lacking for best practices in the clinical validation of machine learning algorithms for both applications produced in laboratories or the verification of software provided by vendors [91,92].

Furthermore, a sizable percentage of the research in the literature demonstrated exceptional predictive power, but only a small number of studies validated their models externally. This could make AI results less sensitive. Overall, data from these trials point to the possibility that these models could be used as supplemental tools to help pathologists and hematologists to diagnose MDS more quickly and affordably. These models, however, have not been sufficiently created yet and tested to take the position of qualified hematologists and pathologists in assessing these samples. Using just one data source to train machine learning models is another issue that these models frequently face [B]. MDS is currently diagnosed using a multimodal approach. Usually, a mix of clinical, PBS, BMS, and FCM data is used [93]. The sensitivity and specificity of the results produced by AI could be increased with appropriate data use and potential integration with human intervention.

Data mutability is another problem with the use of such systems in hematology, and other problems such as insufficient sample sizes and poor study design need to be fixed. Furthermore, a unique problem is overfitting, which is common in machine learning models due to their great degree of data distribution flexibility. This could result in a great fit on the training set but poor performance on the test set.

In addition, selecting the appropriate variables is crucial when developing a model because including unnecessary or irrelevant variables can impair its usefulness. Lastly, for DL to be applied in clinical practice, algorithms that explicitly improve clinical workflow must be created [94]. Repeatability is one of the most challenging issues in image analysis. Determining the role of the technology used in image acquisition and reconstruction can be difficult, since the cognitive processes of an AI agent can be unclear, especially if it is based on an ANN family algorithm. The quality of the hardware, the set and version of reconstruction techniques employed, and certain ambient features that might not be replicable elsewhere can all affect the results.

Finally, using patient data to develop AI solutions raised concerns about data security, privacy, and individual autonomy. In the healthcare industry, autonomy is typically addressed through permission and refers to a person’s freedom to make decisions about their own treatment and personal health information (PHI). Autonomy in AI refers to the use and application of PHI [95]. Large volumes of data, which may be gathered and shared across several organizations, regions, and nations, are necessary for AI programs to learn. Therefore, it is necessary that these data can be shared in a way that respects each person’s privacy and autonomy. People should be aware of who has access to their data, how they are being used, and the possibility that they may be used for secondary purposes or for commercial exploitation beyond the purposes for which they provided their consent [96]. However, medical professionals can improve the precision and effectiveness of detecting MDS by combining AI and ML. Early diagnosis of MDS can be of help in making better decisions and starting earlier treatment, which will enhance patient care.

## Figures and Tables

**Table 1:** Clinical studies on the use of AI in MDS diagnosis.

Material or Technique Employed	Type of Analysis	AI Model	Features of Dataset	Sensitivity and Specificity	Strengths or Weaknesses	Ref.
Peripheral Blood	Morphology	Convolutional neural network-powered deep learning	A highly trained cell image recognition system	93–99.8% 96–100%	Ability to differentiate MDS and Aplastic Anemia	[42]
Bone Marrow	Research of dysplasia	Convolutional neural network-derived machine learning	6453 cell images (c.i.) for training; 806 c.i. for validation: 806 c.i. for testing Inception V3 architecture	90% 99.9%	Difficulty to measure the proportion of dysplasia	[47]
Bone Marrow	Evaluation of reduced granules	Deep neural network	1797 labeled images. Faster R-CNN was trained with ResNet-101 backbone	85.2% 98.9%	Low sensitivity	[48]
Bone Marrow	Morphology	Deep learning model	17,319 annotated cells (development cohort)	Precision 67.4%	Precision inferior to pathologist analysis	[49]
Bone Marrow	Different cellular features	Convolutional neural network	236 samples from 143 MDS subjects; 87 samples from 51 MDS/MPN subjects; 11 healthy controls samples from 11 subjects	- -	WHO subtypes and MDS categories only partially overlapped	[50]
Real-Time Deformability Cytometry	Analysis of dyserythropoiesis through morpho-mechanical pattern	Random forest	Phenotype of BM-derived CD34^+^ HSCs from MDS patients and healthy donors. Seven features were extracted from the contour of each cell and a RF model was trained to distinguish between the healthy state and MDS	Accuracy of 82.9%	The efficiency of CD34 isolation is low	[53]
Flow Cytometry	Hematogone ratio, ratio of CD34+ progenitors	Machine learning	Cohort of 191 patients; external cohort of 89 patients	91.8% 92.5%	Superior sensitivity to Ogata score, prediction of evolution	[57]
Flow Cytometry	Erythroid and myeloid progenitors	Random forest ML	Training cohort 71 MDS, 81 controls; validation cohort 30 MDS, 27 controls; validation cohort 25 MDS with excess of blasts	90% 93%	Processing time less than two minutes	[58]
Flow Cytometry	Analysis of erythropoiesis	Flow-self organizing maps algorithm	Unsupervised clustering analysis; 11 MDS patients	- -	Evidence of subtle erythropoiesis changes	[59]
Flow Cytometry	Detection of binucleated erythroblasts	Convolutional neural network	Bone marrow samples from 14 MDS patients, six ICUS/CCUS patients, six non-MDS controls, and 11 healthy controls	98.2% 78.2%	Increased diagnostic precision	[60]

ICUS: idiopathic cytopenia of undetermined significance; CCUS: clonal cytopenia of undetermined significance.
